# The efficacy and safety of platinum-based chemotherapy for ovarian cancer in pregnancy: A protocol for systematic review and meta-analysis

**DOI:** 10.1097/MD.0000000000031954

**Published:** 2022-11-25

**Authors:** Bei Wang, Yiyi Jia, Liping Liu

**Affiliations:** a Department of Gynaecology, Hebei General Hospital, Shijiazhang, China; b Graduate School, North China University of Science and Technology, Hebei, China.

**Keywords:** chemotherapy, meta-analysis, ovarian cancer, platinum, systematic review

## Abstract

Ovarian cancer during pregnancy is a rare event. Little is known about the treatment of this condition due to lack of high level evidence. Therefore, we performed a protocol for systematic review and meta-analysis to evaluate the efficacy and safety of platinum-based chemotherapy for the treatment of ovarian cancer during pregnancy.

**Methods:**

This systematic review has been registered in PROSPERO (CRD42022370709), which will be conducted in accordance with preferred reporting items for systematic review and meta-analysis protocols 2015 statement. We will search 7 electronic databases to identify relevant studies from inception to October, 2022, which includes PubMed, MEDLINE, Embase, Cochrane Clinical Trials Database, Web of Science, China National Knowledge Infrastructure, and Chinese Biomedical Literature Database. The Cochrane Handbook for systematic reviews of interventions will be performed to assess a broad category of biases in the included studies. The Grading of Recommendations Assessment, Development and Evaluation system will be used to judge the overall quality of evidence supporting outcomes in this work. Data are analyzed with the Review Manager Version 5.3 software.

**Results:**

The results of this meta-analysis would be submitted to peer-reviewed journals for publication.

**Conclusion:**

This paper will provide high-quality synthesis to assess the efficacy and safety of platinum-based chemotherapy for ovarian cancer in pregnancy.

## 1. Introduction

Because of the delay due to planning pregnancy at a later reproductive age and the fact that the frequency of the incidence of numerous neoplasms increases in the fourth decade of life, the number of pregnant women affected by cancer is rising.^[1]^ This challenging problem complicates 1 in 1000 pregnancies and is becoming more common nowadays. Ovarian cancer is the second most common gynecologic cancer diagnosed during pregnancy, complicating 1 in 15,000 to 1 in 32,000 pregnancies.^[2]^ Ovarian cancer represents 3% to 6% of neoplasms and 49% to 75% of ovarian malignancies in pregnancy.^[3]^ According to the statistics, ovarian cancer takes the fifth position among the most common malignancies affecting pregnant women, following breast cancer, thyroid cancer, cervical cancer and Hodgkin lymphoma.^[4,5]^ In 2018, 240,000 new cases were diagnosed, resulting in a rate of incidence of 10 to 15 per 100,000, with the highest mortality rate among female cancers.^[6]^

The treatment goal for pregnant patients with ovarian cancer is the same as for non-pregnant individuals: to improve progression-free survival and preserve fertility.^[7]^ Platinum-based drugs remain as the most active agents in ovarian cancer.^[8]^ In addition, maintaining the optimal balance between management of the mother’s cancer and preserving fetal health is critical.^[9]^ However, studies have shown that while chemotherapy in the second and third trimesters during pregnancy will not increase fetal mortality and deformity, it may increase the incidence of nonmalformation disorders, such as fetal growth restriction, low birth weight, and preterm delivery.^[10]^ Therefore, we performed a protocol for systematic review and meta-analysis to evaluate the efficacy and safety of platinum-based chemotherapy for the treatment of ovarian cancer during pregnancy.

## 2. Methods

### 2.1. Study registration

This systematic review has been registered in PROSPERO (CRD42022370709), which will be conducted in accordance with preferred reporting items for systematic review and meta-analysis protocols 2015 statement.^[11]^ Given that the meta-analysis is a secondary research which based on some previously published data, ethical approval is not necessary for our research.

### 2.2. Inclusion criteria for study selection

#### 2.2.1. Types of study.

To assess the efficacy and safety of platinum-based chemotherapy ovarian cancer during pregnancy, only related randomized controlled trials (RCTs) will be included in the evaluation. Others such as case reports, animal experiments, non-RCTs, or RCT protocol will be excluded.

#### 2.2.2. Types of participants.

The patients range are limited to women diagnosed with primary ovarian cancer during pregnancy, regardless of age, gender, educational status or racial restrictions.

#### 2.2.3. Types of intervention.

Regimens could be composed of platinum chemotherapy alone or platinum compounds plus any other combination of cytotoxic agents. Control arms could be no chemotherapy or any chemotherapy other than platinum-containing regimens.

#### 2.2.4. Types of outcome measures.

The primary outcomes are overall survival and progression-free survival. The secondary outcomes are fetus born weight and complications.

### 2.3. Search strategy

We will search 7 electronic databases to identify relevant studies from inception to October, 2022, which includes PubMed, MEDLINE, Embase, Cochrane Clinical Trials Database, Web of Science, China National Knowledge Infrastructure, and Chinese Biomedical Literature Database. Meanwhile, we will also search Chinese Clinical Trial Registry and ClinicalTrials.gov for ongoing trials with unpublished data. The search strategy in PubMed is shown in Table 1.

**Table 1:** Search strategy (PubMed).

Number	Search terms
#1	gynecological oncology [Ti/Ab]
#2	gynecological tumorsv [Ti/Ab]
#3	ovarian cancer [Ti/Ab]
#4	oophoroma [Ti/Ab]
#5	malignant ovarian tumor [Ti/Ab]
#6	carcinoma of ovary [Ti/Ab]
#7	reproductive system diseases [Ti/Ab]
#8	epithelial ovarian carcinoma [Ti/Ab]
#9	#1 OR #2 OR #3 OR #4 OR #5 OR #6 OR #7 OR #8
#10	chemotherapy [Ti/Ab]
#11	chemotherapeutics [Ti/Ab]
#12	platinum [Ti/Ab]
#13	cisplatin [Ti/Ab]
#14	#10 OR #11 OR #12 OR #13
#15	pregnant [Ti/Ab]
#16	pregnancy [Ti/Ab]
#17	gestation [Ti/Ab]
#18	#15 OR #16 OR #17
#19	#9 AND #14 AND #18

### 2.4. Data collection and analysis

#### 2.4.1. Selection of studies.

**Figure 1.**
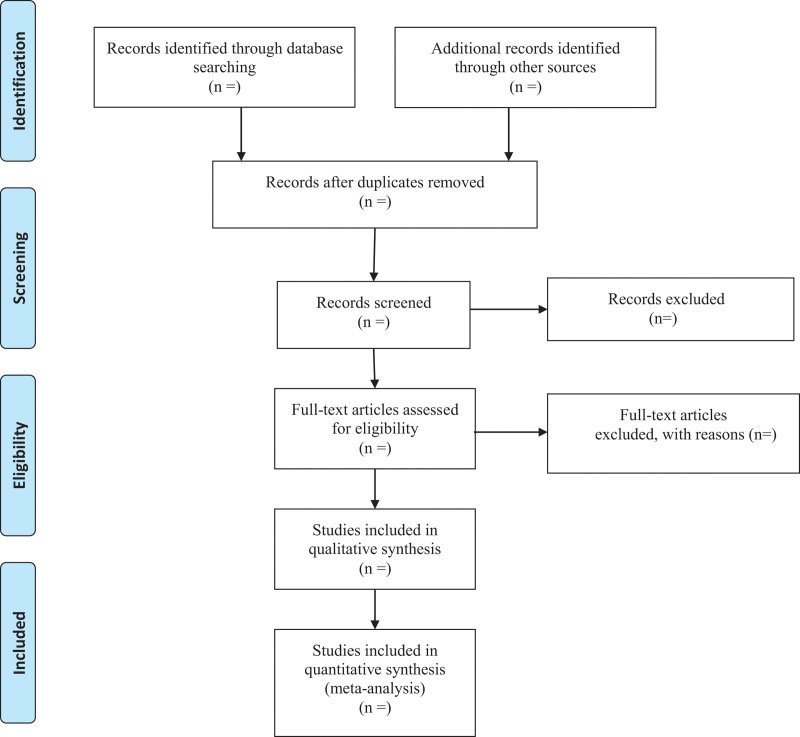
Flow diagram.

Two independent reviewers will screen and evaluate the relevant abstracts and titles of all studies based on pre-defined inclusion criteria and then exclude repetitive or ineligible articles with the reasons. The third investigator will solve any disagreement between the 2 reviewers. The process of screening selection is shown in Figure 1.

#### 2.4.2. Data extraction and management.

Two reviewers will take charge of data extraction and management according to the retrieval strategy, including the title of the study, the journal, the year of publication, the name of the first author, general information, study design, experimental intervention and intervention time, outcomes, and adverse events. If there is any disagreement between 2 reviewers in the data extraction, the group will arbitrate and make decisions together.

#### 2.4.3. Dealing with missing data.

When it comes to missing or unclear data, we will try our best to contact the corresponding author for more detailed information. If it fails, we will analyze it based on available data.

#### 2.4.4. Assessment of risk of bias.

The Cochrane Handbook for systematic reviews of interventions will be performed to assess a broad category of biases in the included studies.^[12]^ We will evaluate biases from the following 7 aspects: random sequence generation, allocation concealment, blinding of the participants and personnel, blinding of the outcome assessments, incomplete outcome data, selective reporting, and other sources of bias. These studies will be assigned as low risk, high risk or unclear risk. Inconsistencies will be resolved by discussion with other reviewers.

#### 2.4.5. Assessment of quality of evidence.

The Grading of Recommendations Assessment, Development and Evaluation system will be used to judge the overall quality of evidence supporting outcomes in this work.^[13]^ And the quality of evidence will be defined as high, moderate, low, or very low.

#### 2.4.6. Measures of treatment effect.

In the study, risk ratios with 95% confidence intervals for analysis will be presented for dichotomous data while standard mean difference or mean difference with 95% confidence intervals will be used for analyzing the continuous data. Data are analyzed with the Review Manager Version 5.3 software.

#### 2.4.7. Assessment of heterogeneity.

This study will use the chi-squared test to calculate the heterogeneity, and the degree of heterogeneity depends on the value of *I*^2^. The results showed that when *I*^2^ > 50%, it will show substantial heterogeneity, and the random effect model will be chosen. If *I*^2^ < 50%, it states no heterogeneity, and the fixed-effect model will be chosen.

#### 2.4.8. Assessment of reporting bias.

If there are over 10 studies included in the meta-analysis, funnel plots will be used to detect the reporting biases.^[14]^

## 3. Discussion

All chemotherapy agents are potentially teratogenic and there is a potential long-term effect on the offspring.^[15]^ The key factor in this situation is the relationship between the doctor’s conscience and the patient’s trust.^[16]^ Decisions concerning the choice of the best management of ovarian cancer are very complex and difficult because of the conflict between the mother’s and the fetus’s wellbeing^[17]^; thus, a multidisciplinary team consisting of an obstetrician, oncologist, pathologist, anesthesiologist, neonatologist and psychologist is mandatory.

Chemotherapy is contraindicated in the first trimester because of the high rate of abortion and abnormal fetal development. Malformations were present in 83.3% of fetuses when chemotherapy was administered during the first trimester; in contrast, malformations have not been reported in most cases in which chemotherapy was administered during the second or third trimesters.^[18]^ This study will summarize current published evidence to provide direct and indirect evidence and provide future direction for studies focused on platinum-based chemotherapy for ovarian cancer in pregnancy.

## Author contributions

**Data analysis and study design:** Liping Liu.

**Language edit:** Yiyi Jia.

**Writing – original draft:** Bei Wang.

## References

[1] Mattiuzzi C Lippi G. Current cancer epidemiology. J Epidemiol Glob Health. 2019;9:217–22. 31854162 10.2991/jegh.k.191008.001 PMC7310786

[2] La Vecchia C. Ovarian cancer: epidemiology and risk factors. Eur J Cancer Prev. 2017;26:55–62. 26731563 10.1097/CEJ.0000000000000217

[3] Gaona-Luviano P Medina-Gaona LA Magana-Perez K. Epidemiology of ovarian cancer. Chin Clin Oncol. 2020;9:47. 32648448 10.21037/cco-20-34

[4] Torre LA Trabert B DeSantis CE. Ovarian cancer statistics, 2018. CA Cancer J Clin. 2018;68:284–96. 29809280 10.3322/caac.21456 PMC6621554

[5] Kroeger PJ Drapkin R. Pathogenesis and heterogeneity of ovarian cancer. Curr Opin Obstet Gynecol. 2017;29:26–34. 27898521 10.1097/GCO.0000000000000340 PMC5201412

[6] Stewart C Ralyea C Lockwood S. Ovarian cancer: an integrated review. Semin Oncol Nurs. 2019;35:151–6. 30867104 10.1016/j.soncn.2019.02.001

[7] Smith ER Borowsky ME Jain VD. Intraperitoneal chemotherapy in a pregnant woman with ovarian cancer. Obstet Gynecol. 2013;122:481–3. 23884267 10.1097/AOG.0b013e31828a845a

[8] McMullen M Karakasis K Madariaga A. Overcoming platinum and PARP-inhibitor resistance in ovarian cancer. Cancers (Basel). 2020;12:1607. 32560564 10.3390/cancers12061607 PMC7352566

[9] Wolters V Amant F. Chemotherapy during pregnancy: careful fetal growth monitoring is mandatory. JCO Oncol Pract. 2020;16:559–60. 32910881 10.1200/OP.20.00628

[10] Zheng X Zhu Y Zhao Y. Taxanes in combination with platinum derivatives for the treatment of ovarian cancer during pregnancy: a literature review. Int J Clin Pharmacol Ther. 2017;55:753–60. 28737125 10.5414/CP202995

[11] Moher D Shamseer L Clarke M. PRISMA-P Group. Preferred reporting items for systematic review and meta-analysis protocols (PRISMA-P) 2015 statement. Syst Rev. 2015;4:1. 25554246 10.1186/2046-4053-4-1 PMC4320440

[12] Higgins JP Altman DG Gotzsche PC. The Cochrane Collaboration’s tool for assessing risk of bias in randomised trials. BMJ. 2011;343:d5928. 22008217 10.1136/bmj.d5928 PMC3196245

[13] Atkins D Best D Briss PA. Grading quality of evidence and strength of recommendations. BMJ. 2004;328:1490. 15205295 10.1136/bmj.328.7454.1490 PMC428525

[14] Deeks JJ Macaskill P Irwig L. The performance of tests of publication bias and other sample size effects in systematic reviews of diagnostic test accuracy was assessed. J Clin Epidemiol. 2005;58:882–93. 16085191 10.1016/j.jclinepi.2005.01.016

[15] Lopez A Rodriguez J Estrada E. Neoadjuvant chemotherapy in pregnant patients with cervical cancer: a Latin-American multicenter study. Int J Gynecol Cancer. 2021;31:468–74. 33649015 10.1136/ijgc-2020-001764

[16] Ngu SF Ngan HY. Chemotherapy in pregnancy. Best Pract Res Clin Obstet Gynaecol. 2016;33:86–101. 26553395 10.1016/j.bpobgyn.2015.10.007

[17] Esposito S Tenconi R Preti V. Chemotherapy against cancer during pregnancy: a systematic review on neonatal outcomes. Medicine (Baltimore). 2016;95:e4899. 27661036 10.1097/MD.0000000000004899 PMC5044906

[18] Picone O Lhomme C Tournaire M. Preservation of pregnancy in a patient with a stage IIIB ovarian epithelial carcinoma diagnosed at 22 weeks of gestation and treated with initial chemotherapy: case report and literature review. Gynecol Oncol. 2004;94:600–4. 15297214 10.1016/j.ygyno.2004.05.030

